# Parallel Monitoring of Glucose, Free Amino Acids, and Vitamin C in Fruits Using a High-Throughput Paper-Based Sensor Modified with Poly(carboxybetaine acrylamide)

**DOI:** 10.3390/bios13121001

**Published:** 2023-11-28

**Authors:** Xinru Yin, Cheng Zhao, Yong Zhao, Yongheng Zhu

**Affiliations:** 1 College of Food Science and Technology, Shanghai Ocean University, Shanghai 201306, China; m210311019@st.shou.edu.cn (X.Y.); allenz_1222@foxmail.com (C.Z.); 2 Henan Railway Food Safety Management Engineering Technology Research Center, Zhengzhou Railway Vocational & Technical College, Zhengzhou 451460, China

**Keywords:** microfluidic paper-based sensor, nanoparticles, glucose, amino acid, vitamin C, colorimetric detection

## Abstract

Herein, a cost-effective and portable microfluidic paper-based sensor is proposed for the simultaneous and rapid detection of glucose, free amino acids, and vitamin C in fruit. The device was constructed by embedding a poly(carboxybetaine acrylamide) (pCBAA)-modified cellulose paper chip within a hydrophobic acrylic plate. We successfully showcased the capabilities of a filter paper-based microfluidic sensor for the detection of fruit nutrients using three distinct colorimetric analyses. Within a single paper chip, we simultaneously detected glucose, free amino acids, and vitamin C in the vivid hues of cyan blue, purple, and Turnbull’s blue, respectively, in three distinctive detection zones. Notably, we employed more stable silver nanoparticles for glucose detection, replacing the traditional peroxidase approach. The detection limits for glucose reached a low level of 0.049 mmol/L. Meanwhile, the detection limits for free amino acids and vitamin C were found to be 0.236 mmol/L and 0.125 mmol/L, respectively. The feasibility of the proposed sensor was validated in 13 different practical fruit samples using spectrophotometry. Cellulose paper utilizes capillary action to process trace fluids in tiny channels, and combined with pCBAA, which has superior hydrophilicity and anti-pollution properties, it greatly improves the sensitivity and practicality of paper-based sensors. Therefore, the paper-based colorimetric device is expected to provide technical support for the nutritional value assessment of fruits in the field of rapid detection.

## 1. Introduction

Fruits, as a kind of highly nutritious food, are considered to be a significant source of carbohydrates, vitamins, amino acids, and dietary fiber in the human diet [1,2]. Consequently, they have assumed a vital role in the dietary guidelines of numerous countries, including China [3]. Gu et al. [4] demonstrated that an increased intake of fruits and vegetables was associated with a reduction in total mortality among Chinese adults. Meanwhile, there is a stronger inverse correlation between fruit intake and mortality. Yuan et al. [5] revealed that the consumption of fruits and vegetables had the potential to counteract the development of hypertension resulting from high fat intake. The abundance of nutrients found in fruits caters to various nutritional requirements of the human body. For instance, glucose intake from fruits can be readily absorbed to provide energy [6], while amino acids play a significant role in regulating bodily functions and enhancing immunity [7]. Moreover, vitamin C, known for its potent antioxidant properties, aids in eliminating excessive free radicals from the body and reducing the risk of cancer. Considering these factors, the nutritional composition of fruits has emerged as a vital criterion for consumers to consider. The detection of these nutrients has been achieved through various methods such as high-performance liquid chromatography (HPLC) [8,9], near-infrared (NIR) spectroscopy [10,11], fluorescence techniques [12,13,14], and liquid chromatography–tandem mass spectrometry (LC/MS) [15,16]. Although these methods offer high sensitivity and accuracy, they involve multiple intricate steps, costly equipment, and skilled personnel, rendering them unsuitable for rapid on-field fruit detection.

Miniaturization, integration, and ease of manipulation are key advantages that have propelled microfluidic devices into the spotlight within the field of application and detection [17,18]. These devices, through the incorporation of grooves or microchannels engraved onto silicon or polymer layers, effectively control fluid direction and reaction, allowing for the integration of multiple reaction steps [19]. In a significant breakthrough, Whitesides’ laboratory reported the development of the first easy-fabricated microfluidic paper-based device (μPAD) setup for chemical analysis in 2007 [20]. The unique feature of this pioneering work was the use of filter paper as a substrate to create a hydrophobic/hydrophilic channel on the paper without any pump or external energy source, relying on capillary action for unpowered fluid transport. This significant breakthrough has made researchers realize that paper served as an outstanding substrate in contemporary applications that prioritize cost-effectiveness, high throughput, and portability [21]. Then, in the realm of paper-based microfluidic devices, various advancements were made in the rapid detection of fruit nutrients. For instance, Akhmad Sabarudin’s research group utilized paper-based platforms to determine the average vitamin C content in four different fruits by leveraging redox reactions [22]. In a similar vein, Siriwan Teepoo et al. constructed a hydrophobic channel with polylactic acid solution to detect sugar levels in sugarcane juice [23], as well as vitamin C levels in beverages [24]. Furthermore, Manas Ranjan Gartia et al. [25] implemented wax printing on Whatman Paper Grade No.1 to analyze the composition and content of fruit juices. Nevertheless, these studies failed to address the limitations of paper in terms of analysis and detection, such as its limited color development effect and inadequate anti-pollution ability. Consequently, it is necessary to incorporate additional materials to adjust and enhance the sensitivity and stability of the paper substrate. 

Zwitterionic polymers have exhibited exceptional properties including high hydrophilicity, long-term durability, resistance to fouling, and environmental stability [26]. Due to their exceptional properties, zwitterionic polymers are grafted onto diverse inorganic/organic surfaces using “graft-from” and “graft-to” strategies, post-zwitterionization, and surface grafting copolymerization methods to fulfill specific application requirements [27]. The negatively charged membranes of Wang et al. were modified by a positively charged zwitterion copolymer through surface adsorption and a cross-linking reaction, which significantly improved the hydrophilicity and antifouling properties of the film surface [28]. Fu et al. enhanced the microfluidic sensor device by incorporating a superhydrophilic zwitterionic polymer. The prepared microfluidic sweat sensor presented exceptional wettability and excellent infusion capacity after modification [29]. 

In this research, a microfluidic paper-based detection platform was constructed via using an acrylic plate as a hydrophobic channel and grafting cellulose filter (CF) with the zwitterionic polymer pCBAA to create a hydrophilic channel. By integrating colorimetry, this platform enabled rapid and simultaneous identification and quantification of three nutrients in fruits with high throughput and low cost. The three analytes underwent enzyme catalysis, ninhydrin reaction, and redox reactions individually within three detection zones on the paper base, resulting in the exhibition of three distinct colors. The concentration of each analyte was then calculated by evaluating the average color intensity of the corresponding detection zone using Image J 1.51 software. Additionally, the pCBAA-μPAD successfully determined glucose, free amino acids, and vitamin C in various fruit samples. The reliability was verified through spectral analysis simultaneously.

## 2. Materials and Methods

### 2.1. Materials and Instruments

Whatman filter paper No. 1 (150 mm diameter) was purchased from Whatman International Ltd. (Shanghai, China). Glucose, vitamin C, and leucine were provided by Sinopharm Chemical Reagent Co., Ltd. (Shanghai, China). β-propiolactone (95%), cuprous(I) bromide (CuBr, 98%), 11-hydroxy-1-undecanethiol (C_11_H_24_OS), 2-bromisobutyryl bromide (BIBB, 98%), 2,2′-bipyridine (BPY), bromoisobutyryl bromide (C_4_H_6_Br_2_O), triethylamine (TEA, 99%), tetrahydrofuran (THF, HPLC grade), chitosan (deacetylation degree > 92%), glucose oxidase (GOx, ≥100 U mg^−1^), ninhydrin, silver nitrate, and 3,3,5,5-tetramethylbenzidine (TMB) were purchased from Sigma-Aldrich (St. Louis, MO, USA). Ethanol (CH_3_CH_2_OH), hydrochloric acid (HCl), ferric chloride (FeCl_3_), sodium borohydride (NaBH_4_), potassium ferricyanide (K_3_[Fe(CN)_6_]), acetone (CH_3_COCH_3_), acetic acid, and sodium acetate were obtained from Aladdin Industrial Inc. (Shanghai, China). Phosphate-buffered saline (PBS) was provided by Solarbio (Beijing, China). All chemical reagents utilized were compounded by ultrapure water without further purification.

### 2.2. Instruments 

The XPS spectra were identified by X-ray photoelectron spectroscopy (XPS, Thermo Scientific EscaLab 250Xi, Waltham, MA, USA). All FT-IR measurement was performed on an FT-IR spectrometer (Thermo Scientific, Waltham, MA, USA). Transmission electron microscopy (TEM) images were captured on a Tecnai G2 T20 electron microscope at 200 kV. The UV–vis spectrum and absorbance were measured using a UV–vis absorption spectrophotometer (Shimadzu Corporation, UV-vis-2550, Kyoto, Japan).

### 2.3. Design and Fabrication of the pCBAA-μPAD 

**Figure 1:**
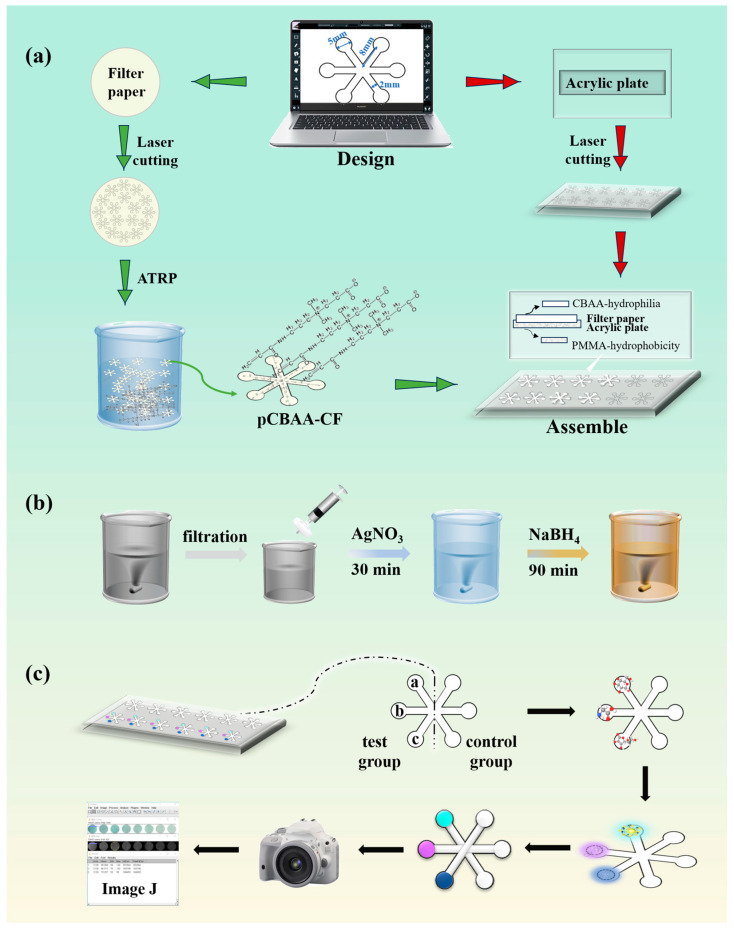
(**a**) Design and production process using a functional poly(carboxybetaine acrylamide)-coated μPAD; (**b**) synthesis of chitosan-stabilized silver nanoparticles; (**c**) the reaction scheme of the pCBAA-μPAD for glucose, vitamin C, and amino acid detection.

The snowflake-shaped pattern, which served as the fundamental design for the μPAD, was created using AutoCAD. The detection zones of this pattern were strategically positioned at the six vertices of a regular hexagon. Each detection area was comprised of a circle with a diameter of 5 mm. Figure 1a illustrate the pCBAA-μPAD, which consisted of two components. Firstly, a hydrophilic paper substrate and a hydrophobic spacer. In the first step, a laser cutting machine was used to carve multiple snowflake-shaped grooves on an acrylic plate composed of poly(methylmethacrylate) (PMMA). This PMMA acrylic plate naturally served as a hydrophobic spacer for the paper-based microfluidic device. Subsequently, in the second step, snowflake-shaped patterns of the same size were laser cut on Whatman filter paper No. 1. Through the surface-initiated atom transfer radical polymerization (SI-ATRP) technique, the synthesized CBAA was successfully grafted onto the cellulose surface, resulting in a hydrophilic paper substrate. The detailed process of the grafting reaction is presented in Figure S1. Finally, the hydrophobic plate and hydrophilic paper substrate were assembled to create a high-throughput pCBAA-μPAD.

### 2.4. Synthesis of Chitosan-Stabilized Silver Nanoparticles 

The preparation of chitosan-stabilized silver nanoparticles (Ch-Ag NPs) was carried out following the method previously described by Yang et al. [30]. As presented in Figure 1b, chitosan (2 mg/mL) was dissolved in a 1% acetic acid solution (200 mL) and stirred for 6 h, followed by filtration through a 0.22 μm microporous filter. Then, 5 mL of 0.01 mol/L silver nitrate solution was mixed with 127.5 mL of the chitosan solution and stirred for 30 min. Then, 2.5 mL of 0.1 mol/L NaBH_4_ solution was rapidly added, and the mixture was further stirred for 90 min. The resulting Ch-Ag NPs exhibited a concentration of 40 mg/L (as Ag) and were stored in a refrigerator at 4 °C away from light.

### 2.5. Simultaneous Colorimetric Detection of Glucose, Free Amino Acids, and Vitamin C

The chromogenic agent in glucose detection was prepared using acetic acid–sodium acetate solution, TMB solution, and Ch-Ag NPs. As shown in Figure 1c, in the detection area a, 1 μL of glucose standard solution and 1 μL of glucose oxidase were first added, followed by 1μL of the configured color developer mixture. The sealing film covered the surface of the sensor and was placed in the oven at 37 °C until the color development completed. Several effective parameters, such as the proportion of each solution, concentration of Ch-Ag NPs, pH value, and reaction time were investigated. For the detection of free amino acids, 1 μL of leucine standard solution, 1 μL of acetic acid buffer solution and 1 μL of ninhydrin were added to the detection area b successively and placed in the oven at 60 °C. The parameters of pH value, concentration, temperature, and time were optimized experimentally. The reactive chromogenic agent of vitamin C was composed of FeCl_3_, K_3_[Fe(CN)_6_], and HCl. Next, 1 μL of vitamin C standard solution and 1 μL of chromogenic agent were added to the detection area c successively, and a series of adjustments were made to the concentration and proportion of each solution in the mixed chromogenic agent. The optimal detection scheme after optimization of the three target detection objects is described in detail in the Supplementary Material.

After the completion of three colorimetric reactions, image acquisition (presented in the Materials and Methods section of the Supplementary Material) was performed promptly. The acquired images were analyzed and compared for color intensity using Image J 1.51 software. This analysis established a linear relationship between the concentrations of glucose, free amino acids, and vitamin C and the average relative intensity of grayscale. This relationship was subsequently utilized for qualitative and quantitative analysis of actual samples.

### 2.6. Sample Preparation

Thirteen fruit varieties were purchased from the local market and used as test objects to assess the performance of the sensor. The fruits were subjected to pretreatment based on the Chinese National Standard system (GB 5009.8-2016 and GB 5009.86-2016). The fruit was washed, dried, peeled, and cored, then cut into small pieces. Approximately 30 g of the fruit sample was weighed for homogenization. The homogenate was subjected to ultrasonic extraction in an ice bath for 5 min, followed by centrifugation at 6000 rpm for 15 min to obtain the supernatant. The extracted supernatant was further filtered using a sterile microporous ultrafiltration membrane (0.22 μm) and stored in a refrigerator at 4 °C for future use. All fruit samples used in the experiment were freshly purchased.

## 3. Results

### 3.1. FT-IR and XPS Analysis 

**Figure 2:**
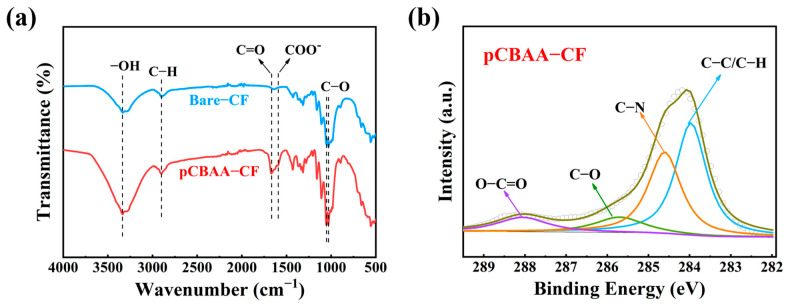
(**a**) The FT−IR spectra of bare−CF and pCBAA−CF; (**b**) representative XPS high−resolution C1s spectra of pCBAA−CF.

The grafting results on the paper substrate were confirmed using FT-IR and XPS analysis. As depicted in Figure 2a, the bare-CF spectrum exhibited characteristic peaks of cellulose paper, including the broad stretching vibration of -OH at 3337 cm^−1^, the skeletal vibration of -CH_2_- at 2900 cm^−1^, and the skeletal stretching vibrations of C-O at 1055 cm^−1^ and 1026 cm^−1^ [31]. In the FT-IR spectrum of pCBAA-CF, characteristic absorption peaks of pCBAA were observed in addition to the presence of characteristic peaks of cellulose paper. These included the vibrational stretching of the C=O bond at 1662 cm^−1^ [32] and the asymmetric stretching of the COO^−^ group at 1585 cm^−1^ [33]. 

As shown in Figure S2, pCBAA-CF revealed the presence of carbon and oxygen elements, similar to bare-CF, as well as the detection of nitrogen element specific to pCBAA. The high-resolution XPS C 1s spectrum of bare-CF was fitted with two peaks centered at 284.6 and 286.3 eV, corresponding to C-C/C-H and C-O bonds [31], respectively (Figure S3). In Figure 2b, besides the binding energy observed in bare-CF, pCBAA-CF displayed two additional signals at 285.2 and 288.6 eV, respectively assigned to C-N and O-C=O groups [34]. Thus, all above results confirm that the pCBAA was successfully grafted onto bare-CF using the ATRP polymerization method.

### 3.2. Properties Characterization of pCBAA-μPAD 

**Figure 3:**
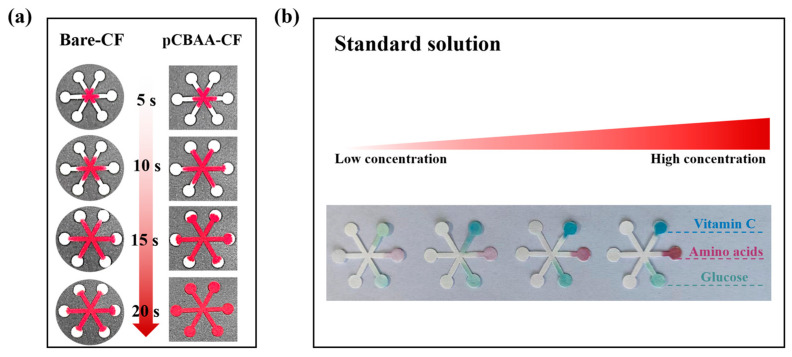
(**a**) Time dependence of red ink flow rate on bare-CF and pCBAA-CF; (**b**) gradient sensing performance of glucose, amino acids, and vitamin C on pCBAA-CF.

To verify the hydrophilic performance of pCBAA-μPADs, an equal amount of red ink was simultaneously dropped onto the surface of pre-modified and post-modified paper substrates. Then, the liquid flow time on both surfaces was compared. As shown in Figure 3a, the red ink on pCBAA-CF completely covered the entire paper platform within 20 s, while on the unmodified bare-CF, it did not reach the full coverage. This demonstrated that pCBAA significantly increased the hydrophilicity of the paper substrate, accelerating the capillary action of the detection liquid and thus shortening the detection time. The colorimetric sensing performance of the pCBAA-μPAD is further revealed in Figure 3b. While the concentrations of glucose, amino acids, and vitamin C increased, the corresponding color depth and grayscale values gradually increased. It was confirmed that pCBAA-functionalized paper-based platforms exhibited significant detection performance for various target nutrients.

### 3.3. Detection of Glucose

**Figure 4:**
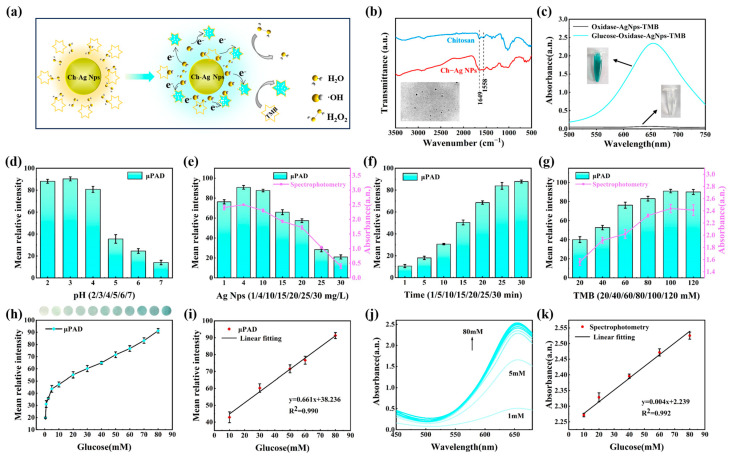
(**a**) Principle of glucose detection using Ch-Ag NPs; (**b**) FTIR spectrum of Ch-Ag NPs and chitosan; (**c**) UV absorbance of experimental group and blank control group; (**d**) effect of pH on detection method; (**e**) effect of Ch-Ag NP concentration on enzymatic activity; (**f**) effect of time on reaction progress; (**g**) effect of TMB concentration on colorimetric reaction; (**h**) effect of different glucose concentrations on average grayscale intensity; (**i**) linear relationship between glucose concentrations and average grayscale intensity; (**j**) effect of different glucose concentrations on UV absorbance; (**k**) correlation between glucose concentration and UV absorbance.

The detection of glucose was achieved through the catalytic activity of chitosan–silver nanoparticle (Ch-Ag NP) peroxidase. Glucose was first decomposed by glucose oxidase into hydrogen peroxide (H_2_O_2_) and gluconic acid. H_2_O_2_ molecules adsorb on Ch-Ag NP surfaces, generating hydroxyl radicals (·OH). Hydroxyl radicals reacted with colorless TMB, resulting in the formation of the TMB diamine oxidized in a blue form, as shown in Figure 4a [35,36]. Ch-Ag NPs exhibited excellent catalytic ability in the presence of H_2_O_2_, and the presence of chitosan increased the stability and storage of silver nanoparticles [37]. In order to demonstrate the successful combination of chitosan and silver nanoparticles, a comparison of FT-IR analysis was conducted for chitosan and Ch-Ag NPs. As shown in Figure 4b, characteristic peaks of chitosan appeared in Ch-Ag NPs, with the peak around 1649 cm^−1^ attributed to the stretching vibration of the C=O bond (amide I), and the peak at 1559 cm^−1^ was enhanced with the addition of chitosan [38]. The synthesized Ch-Ag NPs were spherical with a diameter of approximately 12 nm (Figure S4).

The cyan blue product showed a maximum absorption peak at 650 nm. In contrast, no significant color change was observed in the blank reaction system without the addition of glucose (Figure 4c). The influence of different pH environments on catalytic reaction was firstly investigated through grayscale intensity analysis. Ch-Ag NPs presented strong activity within a relatively wide pH range of 2–4. Then, pH = 3 was selected as the optimal reaction environment for subsequent catalytic reaction kinetics (Figure 4d). Subsequently, the effect of Ch-Ag NP concentration on peroxidase activity was explored. As observed in Figure 4e, the grayscale intensity was significantly higher when Ch-Ag NPs were diluted in acetic acid buffer at concentrations of 4–10 mg/L. Considering the characteristic absorption at 650 nm, Ch-Ag NPs exhibited the strongest enzymatic activity at a concentration of 4 mg/L. The catalytic reaction was time-dependent, as shown in Figure 4f, where the production of oxidized TMB (oxTMB) and the corresponding grayscale intensity increased with prolonged reaction time. After 30 min, the average grayscale intensity of oxTMB tended to stabilize. Similarly, the concentration of TMB also affected the colorimetric reaction. TMB concentrations between 60 and 120 mmol/L demonstrated higher grayscale intensity and UV absorbance (Figure 4g). A TMB concentration of 100 mmol/L was chosen for the preparation of the reaction color reagent. Under the optimal conditions, as revealed in Figure 4h,i, there was a good linear relationship (y = 0.661x + 38.236, R^2^ = 0.990) between glucose concentration and the average grayscale intensity of the pCBAA-μPAD within the range of 10–80 mmol/L. Meanwhile, the detection limit (*LOD*) was as low as 0.049 mmol/L.

(1) $$LOD=\frac{3\sigma }{S}$$

The value of *LOD* is calculated using Equation (1), where *σ* represents the standard deviation of the blank sample (*n* = 11), and *S* refers to the slope of the fitted standard curve. A comparative experiment using spectrophotometry showed a good linear equation (y = 0.004x + 2.239, R^2^ = 0.992) for the data collected at the characteristic peak of 650 nm (Figure 4j,k). These results demonstrated that there was good synchronization between the two methods. Thus, the pCBAA-μPAD offers the advantages of a microfluidic device, providing a more convenient and rapid approach for glucose detection.

### 3.4. Detection of Free Amino Acids

**Figure 5:**
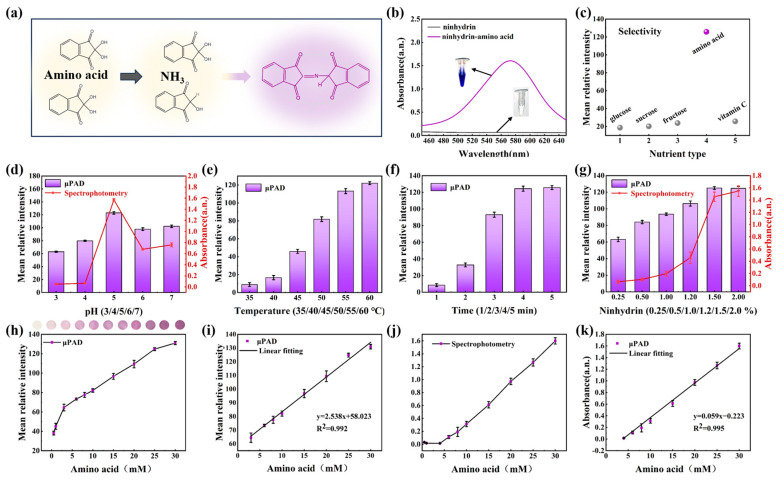
(**a**) Principle of free amino acid detection; (**b**) UV absorbance of experimental group and blank control group; (**c**) selective testing of detection method; (**d**) effect of pH on detection method; (**e**) effect of temperature on color reaction; (**f**) effect of time on reaction progress; (**g**) effect of ninhydrin concentration on colorimetric reaction; (**h**) effect of different amino acid concentrations on average grayscale intensity; (**i**) linear relationship between amino acid concentrations and average grayscale intensity; (**j**) effect of different amino acid concentrations on UV absorbance; (**k**) correlation between amino acid concentration and UV absorbance.

The reaction principle between free amino acids and ninhydrin was depicted in Figure 5a. Amino acids produced carbon dioxide, ammonia, and aldehyde by oxidation, while ninhydrin hydrate was reduced to its reduced form. Subsequently, the generated ammonia and reductive ninhydrin with another molecule of ninhydrin hydrated, forming a purple compound [39]. This purple compound exhibited maximum absorbance at 570 nm, and the UV absorbance of the blank control group without amino acids remained almost unchanged (Figure 5b). To evaluate the selectivity of ninhydrin for amino acid detection on the pCBAA-μPAD, potential interfering compounds such as glucose, sucrose, fructose, and vitamin C were introduced in the sample zone. All experiments were conducted with the same concentration of 30 mmol/L. The results in Figure 5c show that there was no significant change in the grayscale intensities of all interfering compounds, while the average intensity of amino acids was considerably high. Consequently, the developed pCBAA-μPAD presented high specificity for free amino acids.

The pH, temperature, time, and concentration of ninhydrin on the pCBAA-μPAD were carefully optimized to achieve the optimal color reaction. Adjustments were made to the pH value, ranging from 3 to 7, in order to determine the ideal solution environment for achieving the best coloring effect. The results, as revealed in Figure 5d, indicated that the average relative intensity on the paper-based platform reached its peak at pH = 5. The impact of temperature on the reaction was also explored. As depicted in Figure 5e, temperatures below 40 °C led to minimal purple color formation and insignificant grayscale intensity. To minimize reaction time, the optimal reaction temperature was determined to be 60 °C, with the color of the system stabilizing after 5 min (Figure 5f). Ninhydrin, a key component of the color reaction, had its optimal concentration determined by analyzing grayscale intensity and UV absorbance, as illustrated in Figure 5g. The average relative intensity displayed an increasing trend with rising ninhydrin concentration, stabilizing at 1.5%. Consequently, all the aforementioned optimized conditions were utilized in pretreating the detection area of the pCBAA-μPAD, as demonstrated in Figure 5h,i. The relationship between amino acid concentration and average grayscale intensity exhibited good linearity within the range of 3–30 mmol/L, with a correlation equation of y = 2.538x + 58.023 and a correlation coefficient of R^2^ = 0.992. The low detection limit was determined to be 0.236 mmol/L, which was calculated by Equation (1). Furthermore, the method was validated using UV spectrophotometry. It is revealed in Figure 5j that there is a positive linear relationship between absorbance at 570 nm and amino acid concentration (y = 0.059x − 0.223, R^2^ = 0.995). The consistency between the outcomes obtained from the pCBAA-μPAD and spectrophotometry underscores the vast potential value of paper-based microfluidics in the analysis and detection of fruit nutrients.

### 3.5. Detection of Vitamin C 

**Figure 6:**
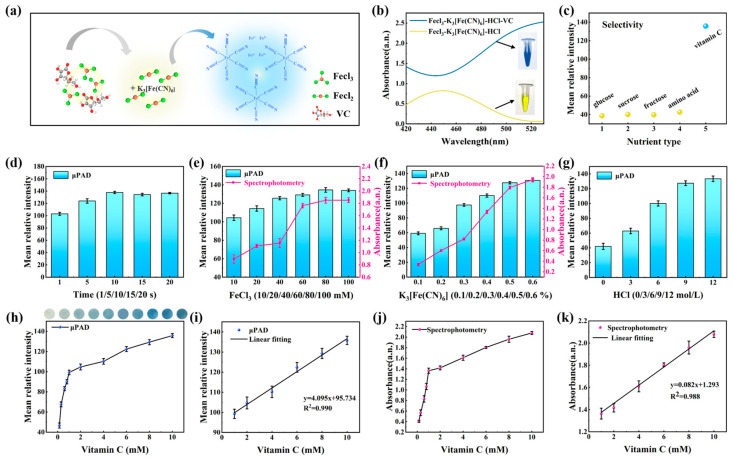
(**a**) Principle of vitamin C detection; (**b**) UV absorbance of experimental group and blank control group; (**c**) selective testing of detection method; (**d**) effect of time on reaction progress. (**e**) Effect of Fecl_3_ concentration on detection method; (**f**) effect of K_3_[Fe(CN)_6_] concentration on colorimetric reaction; (**g**) effect of HCl concentration on detection method; (**h**) effect of different vitamin C concentrations on average grayscale intensity; (**i**) linear relationship between vitamin C concentrations and average grayscale intensity; (**j**) effect of different vitamin C concentrations on UV absorbance; (**k**) correlation between vitamin C concentration and UV absorbance.

The detection principle of vitamin C was based on its strong reducing ability. As shown in Figure 6a, the Fe^3+^ in FeCl_3_ was reduced to Fe^2+^, and a specific color reaction between potassium ferricyanide and divalent iron formed a coordination compound called Turnbull’s blue [40]. The quantity of Turnbull’s blue generated was reflected by the grayscale intensity, establishing the relationship between the concentration of vitamin C and the average grayscale intensity. From the ultraviolet absorbance values presented in Figure 6b, it was observed that the color of Turnbull’s blue in the experimental group was more intense compared to the blank group without the addition of vitamin C, providing a solid foundation for colorimetric sensing on the pCBAA-μPAD. Given the diverse range of nutrients in fruits, it was essential to study the selectivity of this reaction. As observed in Figure 6c, in the presence of various interfering substances in fruits, only vitamin C exhibited strong coloration and the highest grayscale intensity. This specificity was an important requirement for analysis and detection using the pCBAA-μPAD.

In order to achieve a stable response of the pCBAA-μPAD, several important parameters were investigated in the experiment. Due to the rapid reaction between divalent iron and potassium ferricyanide, as shown in Figure 6d, the reaction was completed in almost one second and reached a steady state within 10 s. The colorimetric reagent was prepared from FeCl_3_, K_3_[Fe(CN)_6_], and HCl, so the concentrations of these three reagents were carefully optimized. FeCl_3_ served as a key mediator in this reaction, and optimization was conducted based on both the average grayscale intensity and the ultraviolet absorbance, as shown in Figure 6e. From the results displayed on the pCBAA-μPAD, FeCl_3_ revealed good grayscale values at concentrations above 60 mmol/L. Combining with UV spectrophotometry, 100 mmol/L FeCl_3_ was chosen for the preparation of the colorimetric reagent mixture. Another important parameter, K_3_[Fe(CN)_6_], when dissolved in deionized water, produced a yellow solution. If the concentration was too high, it could hinder the grayscale information processing of Turnbull’s blue. Therefore, a concentration of 0.5% K_3_[Fe(CN)_6_] was selected for the preparation of the colorimetric reagent mixture (Figure 6f). Furthermore, the results for HCl with concentrations ranging from 0 to 12 mol/L indicated that a strong acid environment promoted the progress of the reaction. Therefore, 12 mol/L HCl was chosen as the optimal condition (Figure 6g). Ultimately, the colorimetric reagent was prepared by combining 100 mmol/L FeCl_3_, 0.5% K_3_[Fe(CN)_6_] and 12 mol/L HCl in a 3:2:1 ratio. When vitamin C interacted with the colorimetric reagent on the pCBAA-μPAD, the grayscale intensity enhanced with the increase in vitamin C concentration, showing a good linear response within the range of 1–10 mmol/L. The correlation equation was y = 4.095x + 95.734, with an R^2^ value of 0.990 (Figure 6h,i). According to Equation (1), the detection limit for vitamin C was determined to be 0.125 mmol/L. Similarly, by collecting absorbance data of Turnbull’s blue at 500 nm, a good linear equation was established (y = 0.082x + 1.293, R^2^ = 0.988) (Figure 6j,k). These results demonstrated that the pCBAA-μPAD exhibited excellent sensing performance and has good detection capabilities for vitamin C.

### 3.6. Real Samples Study

**Figure 7:**
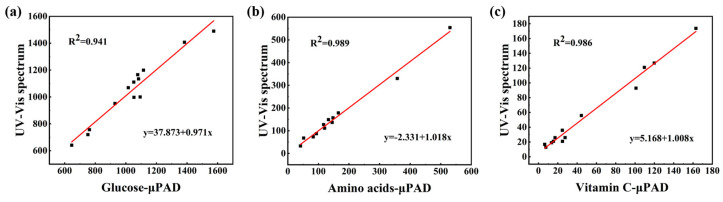
Linear regression analysis of μPAD and UV spectrophotometry. (**a**) Glucose; (**b**) amino acids; (**c**) vitamin C.

The detection performance of the pCBAA-μPAD was further evaluated by analyzing the nutrient content in 13 common real fruit samples (such as peach, grape, pear, and kiwi). The obtained results were compared with those obtained using UV spectrophotometry. From Table 1, Table 2 and Table 3, it was observed that there was no difference between the concentrations of glucose, free amino acids, and vitamin C obtained by the pCBAA-μPAD and those obtained by UV spectrophotometry. In order to verify the correlation between the two methods, regression analysis was conducted on each group of test results, as shown in Figure 7. UV spectrophotometry was positively correlated with the μPAD in glucose with r = 0.970, where the regression equation was significant with R^2^ = 0.941 and *p* < 0.001. Meanwhile, UV spectrophotometry was positively correlated with the μPAD in amino acids and vitamin C, and the regression equation was significant with R^2^ values of 0.989 and 0.986, respectively. Similarly, the *p* value of both analyses was less than 0.001. Thus, the feasibility of the paper-based sensing device for rapid detection of fruit nutrients was confirmed. Additionally, compared to UV spectrophotometry, the proposed pCBAA-μPAD required less analysis time and reagent consumption, which were also enabling efficient detection of three nutrients simultaneously. These results revealed that the sensors had excellent advantages and held great value for routine testing of fruit products.

## 4. Discussion

In recent years, numerous research groups have been actively searching for new breakthroughs in the detection methods of glucose, amino acids, and vitamin C. As represented in Table 4, there are comparative analyses of these methods. Filiz et al. [41] designed a stable electrospun nanofiber composed of chitosan (CS) and polyvinyl alcohol (PVA). They utilized UV–visible spectrophotometry for colorimetric detection of glucose in aqueous media. This method exhibited strong stability, but its accuracy needed further improvement. Georgelis et al. [42] combined modern HPLC systems with high-precision mass spectrometers (HPLC-MS) to rapidly determine multiple sugars in mature potato tubers and strawberry fruits. This approach offered high accuracy and sensitivity. However, the equipment cost was high, and the operation was complex. Su et al. [43] developed three methods for the determination of small molecule carbohydrates in jujube extracts: high-performance liquid chromatography–evaporative light scattering detection (HPLC-ELSD), liquid chromatography–electrospray ionization tandem mass spectrometry (LC-ESI-MS/MS), and gas chromatography–mass spectrometry (GC-MS). HPLC-ELSD and LC-ESI-MS/MS presented high accuracy, which was suitable for quantitative analysis, but they required a skilled operator for assessment. GC-MS is more suitable for qualitative analysis. The detection of amino acids mainly relies on UV–vis spectrophotometry [44], near-infrared (NIR) [45], and HPLC-MS [46] methods. According to these studies, UV–vis spectrophotometry was simple to operate but has moderate accuracy. NIR was fast but not suitable for dispersed sample systems. The HPLC-MS combination offered high sensitivity but was not operationally convenient. Shrivas [47] developed reverse-phase high-performance liquid chromatography (RP-HPLC) with a diode array detector (DAD) for vitamin detection. Paper spray mass spectrometry (PS-MS) [48] and electroanalysis [49] were effective methods for detecting vitamin C content.

In comparison to the various detection methods mentioned above, the as-prepared pCBAA-μPAD in this study enabled parallel detection of glucose, amino acids, and vitamin C. The advantages of this method included high efficiency, good selectivity, extremely low cost, strong portability, simple operation, and short duration. Thus, it is suitable for real-time on-site testing, which can complement the inconveniences of large analytical instruments.

## 5. Conclusions

In this study, a simple and rapid paper-based sensing strategy was developed for the simultaneous determination of glucose, free amino acids, and vitamin C in fruits. The fabrication of the pCBAA-μPAD was cost-effective and easily assembled without external devices. Detection of the three analytes was achieved through colorimetric reactions using corresponding selective substrates on the paper-based platform. After optimizing the experimental conditions, the detection limits for glucose, free amino acids, and vitamin C on the paper-based sensing platform were determined to be 0.049 mmol/L, 0.236 mmol/L, and 0.125 mmol/L, respectively. Through comparison between the evaluation of the nutritional composition from actual fruit samples by the paper-based platform and UV spectrophotometry, the feasibility of the method was validated. These findings reveal the outstanding application potential of the developed paper-based sensor for rapid and routine analysis of nutrients in fruit products, which is closely related to fruit quality control.

## Figures and Tables

**Table 1:** The detection of glucose in fruit samples with pCBAA-μPAD and UV–visible spectrophotometry.

Samples	Glucose (mg/100 g)		RSD (%) (*n* = 3)	
	μPAD	UV–vis Spectrum	μPAD	UV–vis Spectrum
Yellow peach	762.284	756.668	2.6	2.5
Xinyi peach	646.173	640.763	2.1	3.0
Longquan peach	928.909	950.763	3.1	3.1
Yangshan peach	752.196	719.653	3.9	2.5
Jasmine grapes	1053.776	996.987	2.6	2.9
Sunshine grapes	1383.334	1406.763	2.9	2.5
Kiwi fruit	1574.912	1489.866	3.5	2.9
Litchi	1077.419	1165.763	3.1	2.3
Sugar pear	1083.361	1134.862	2.9	2.5
Crown pear	1115.616	1198.765	2.9	3.1
Su Crisp pear	1016.362	1068.762	2.2	2.9
Mangosteen	1094.019	999.605	2.1	2.4
Longan	1051.619	1110.384	3.2	3.1

**Table 2:** The detection of free amino acids in fruit samples with pCBAA-μPAD and UV–visible spectrophotometry.

Samples	Amino Acids (mg/100 g)		RSD (%) (*n* = 3)	
	μPAD	UV–vis Spectrum	μPAD	UV–vis Spectrum
Yellow peach	165.217	178.753	2.9	3.4
Xinyi peach	41.425	32.7527	4.5	2.9
Longquan peach	133.072	148.763	3.9	4.2
Yangshan peach	120.271	110.763	2.9	2.7
Jasmine grapes	144.995	136.7652	2.4	3.8
Sunshine grapes	147.597	156.7573	3.5	2.7
Kiwi fruit	51.811	67.753	4.3	3.5
Litchi	357.854	329.7653	3.8	2.3
Sugar pear	82.969	73.762	2.5	2.6
Crown pear	82.969	72.656	3.4	4.1
Su Crisp pear	93.355	86.767	4.2	4.3
Mangosteen	117.103	126.763	3.7	2.9
Longan	530.310	553.763	3.9	3.7

**Table 3:** The detection of vitamin C in fruit samples with pCBAA-μPAD and UV–visible spectrophotometry.

Samples	Vitamin C (mg/100 g)		RSD (%) (*n* = 3)	
	μPAD	UV–vis Spectrum	μPAD	UV–vis Spectrum
Yellow peach	24.810	20.876	3.5	2.7
Xinyi peach	101.035	92.878	4.2	3.8
Longquan peach	15.451	20.863	2.6	3.5
Yangshan peach	44.363	55.864	3.8	3.2
Jasmine grapes	24.6115	35.763	4.1	3.1
Sunshine grapes	7.608	12.733	2.6	2.3
Kiwi fruit	162.976	173.763	4.3	2.6
Litchi	119.783	126.733	2.5	2.4
Sugar pear	6.386	16.733	4.2	2.8
Crown pear	13.481	18.873	3.5	3.1
Su Crisp pear	17.147	25.763	3.6	4.0
Mangosteen	27.532	25.863	2.9	2.5
Longan	109.637	120.863	3.8	2.8

**Table 4:** Comparison of detection methods for glucose, amino acids, and vitamin C.

Method	Target	LOD	Characteristic	Path	Ref.
UV spectrophotometry	Glucose	2.70 mM	Strong stability, insufficient accuracy	CS/PVA	[41]
HPLC/MS		0.10 ng	High accuracy, complex operation	-	[42]
HPLC-ELSD		1.03 μg/mL	Good repeatability, moderate sensitivity	-	[43]
LC–ESI–MS/MS		0.01 μg/mL	High sensitivity, high accuracy		
GC–MS		0.65 μg/mL	Moderate accuracy, qualitative analysis		
UV spectrophotometry	Amino acids	0.15 μM	Strong stability, insufficient accuracy	AgNPs	[44]
NIR		52 nM	High sensitivity, simple operation.	-	[45]
HPLC-FLD-MS/MS		0.13–1.13 nM	High sensitivity, expensive instrument	-	[46]
RP-HPLC	Vitamin C	0.1 μg/mL	High accuracy, complex operation	DAD	[47]
PS-MS		0.3 μg/mL	Moderate accuracy, short duration.	-	[48]
Electroanalysis		0.067 μM	High accuracy, high sensitivity	SO_2_NPs	[49]
pCBAA-μPAD	Three analytes	0.049/0.236/0.125 mM	High accuracy, portable, simple operation	Paper sensor	This work

## Data Availability

The data presented in this study are available on request from the corresponding author.

## Supplementary Materials

The following supporting information can be downloaded at: https://www.mdpi.com/article/10.3390/bios13121001/s1. Figure S1: Reaction scheme for grafting pCBAA onto CF [50,51]; Figure S2: The XPS spectra of bare-CF and pCBAA-CF; Figure S3: Representative XPS high-resolution C1s spectra of bare-CF; Figure S4: TEM image of Ch-Ag NPs; Equation (S1): Δ*R* = $\bar{R}$*_after_* − $\bar{R}$*_before_*; Equation (S2): Δ*G* = $\bar{G}$*_after_* − $\bar{G}$*_before_*; Equation (S3): Δ*B* = $\bar{B}$*_after_* − $\bar{B}$*_before_*; Equation (S4): Δ*Gray* = 0.30Δ*R* + 0.59Δ*G* + 0.11Δ*B*.

## References

[1] Sui Kiat C., Alasalvar C., Shahidi F. (2019). Superfruits: Phytochemicals, antioxidant efficacies, and health effects-a comprehensive review. Crit. Rev. Food Sci. Nutr..

[2] Ho K., Ferruzzi M.G., Wightman J.D. (2020). Potential health benefits of (poly)phenols derived from fruit and 100% fruit juice. Nutr. Rev..

[3] Mason-D’Croz D., Bogard J.R., Sulser T.B., Cenacchi N., Dunston S., Herrero M., Wiebe K. (2019). Gaps between fruit and vegetable production, demand, and recommended consumption at global and national levels: An integrated modelling study. Lancet Planet. Health.

[4] Gu Y.X., He Y.S., Ali S.H., Harper K., Dong H.J., Gittelsohn J. (2021). Fruit and vegetable intake and all-cause mortality in a Chinese population: The China health and nutrition survey. Int. J. Environ. Res. Public Health.

[5] Yuan S., Yu H.J., Liu M.W., Tang B.W., Zhang J., Gasevic D., Larsson S.C., He Q.Q. (2020). Fat intake and hypertension among adults in China: The modifying effects of fruit and vegetable intake. Am. J. Prev. Med..

[6] Lin P.H., Sheu S.C., Chen C.W., Huang S.C., Li B.R. (2022). Wearable hydrogel patch with noninvasive, electrochemical glucose sensor for natural sweat detection. Talanta.

[7] Lutt N., Brunkard J.O. (2022). Amino acid signaling for TOR in eukaryotes: Sensors, transducers, and a sustainable agricultural fuTORe. Biomolecules.

[8] Shen S., Du L.N., Hu X.B. (2020). Determiination of vitamin C in hordeum vulgare L. Seedling powder by HPLC. Fresenius Environ. Bull..

[9] Serafim J.A., Silveira R.F., Vicente E.F. (2021). Fast determination of short-chain fatty acids and glucose simultaneously by ultraviolet/visible and refraction index detectors via high-performance liquid chromatography. Food Anal. Meth..

[10] Guelpa A., Marini F., du Plessis A., Slabbert R., Manley M. (2017). Verification of authenticity and fraud detection in South African honey using NIR spectroscopy. Food Control.

[11] Li L., Hu D.Y., Tang T.Y., Tang Y.L. (2023). Non-destructive detection of the quality attributes of fruits by visible-near infrared spectroscopy. J. Food Meas. Charact..

[12] del Barrio M., Cases R., Cebolla V., Hirsch T., de Marcos S., Wilhelm S., Galban J. (2016). A reagentless enzymatic fluorescent biosensor for glucose based on upconverting glasses, as excitation source, and chemically modified glucose oxidase. Talanta.

[13] Du R.R., Yang D.T., Jiang G.J., Song Y.R., Yin X.Q. (2020). An approach for in situ rapid detection of deep-sea aromatic amino acids using laser-induced fluorescence. Sensors.

[14] Lu Q.J., Chen X.G., Liu D., Wu C.Y., Liu M.L., Li H.T., Zhang Y.Y., Yao S.Z. (2019). A turn-on fluorescent probe for vitamin C based on the use of a silicon/CoOOH nanoparticle system. Microchim. Acta.

[15] Asanica A.C., Catana L., Catana M., Burnete A.G., Lazar M.A., Belc N., Sanmartin A.M. (2019). Internal validation of the methods for determination of water-soluble vitamins from frozen fruits by HPLC-HRMS. Rom. Biotech. Lett..

[16] Harada M., Karakawa S., Yamada N., Miyano H., Shimbo K. (2019). Biaryl axially chiral derivatizing agent for simultaneous separation and sensitive detection of proteinogenic amino acid enantiomers using liquid chromatography-tandem mass spectrometry. J. Chromatogr. A.

[17] Tao Y.Z., Shen H.C., Deng K.Y., Zhang H.M., Yang C.Y. (2021). Microfluidic devices with simplified signal readout. Sens. Actuator B-Chem..

[18] Wu K.M., He X.L., Wang J.L., Pan T., He R., Kong F.Z., Cao Z.M., Ju F.Y., Huang Z., Nie L.B. (2022). Recent progress of microfluidic chips in immunoassay. Front. Bioeng. Biotechnol..

[19] Cui P., Wang S.C. (2019). Application of microfluidic chip technology in pharmaceutical analysis: A review. J. Pharm. Anal..

[20] Martinez A.W., Phillips S.T., Butte M.J., Whitesides G.M. (2007). Patterned paper as a platform for inexpensive, low-volume, portable bioassays. Angew. Chem. Int. Ed..

[21] Wen G., Guo Z.G. (2020). A paper-making transformation: From cellulose-based superwetting paper to biomimetic multifunctional inorganic paper. J. Mater. Chem. A.

[22] Andini, Andayani U., Anneke, Sari M.I., Sabarudin A. IOP Printed Low-Cost Microfluidic Paper-based Analytical Devices for Quantitative Detection of Vitamin C in Fruits. Proceedings of the 9th Annual Basic Science International Conference (BaSIC)—Recent Advances in Basic Sciences Toward 4.0 Industrial Revolution.

[23] Aksorn J., Teepoo S. (2020). Development of the simultaneous colorimetric enzymatic detection of sucrose, fructose and glucose using a microfluidic paper-based analytical device. Talanta.

[24] Kaewchuay N., Jantra J., Khettalat C., Ketnok S., Peungpra N., Teepoo S. (2021). On-site microfluidic paper- based titration device for rapid semi-quantitative vitamin C content in beverages. Microchem. J..

[25] Prasad A., Tran T., Gartia M.R. (2019). Multiplexed paper microfluidics for titration and detection of ingredients in beverages. Sensors.

[26] Laschewsky A., Rosenhahn A. (2019). Molecular design of zwitterionic polymer interfaces: Searching for the difference. Langmuir.

[27] Li D.X., Wei Q.L., Wu C.X., Zhang X.F., Xue Q.H., Zheng T.R., Cao M.W. (2020). Superhydrophilicity and strong salt-affinity: Zwitterionic polymer grafted surfaces with significant potentials particularly in biological systems. Adv. Colloid Interface Sci..

[28] Wang S.Y., Fang L.F., Matsuyama H. (2020). Construction of a stable zwitterionic layer on negatively-charged membrane via surface adsorption and cross-linking. J. Membr. Sci..

[29] Fu F.F., Wang J.L., Tan Y.R., Yu J. (2022). Super-hydrophilic zwitterionic polymer surface modification facilitates liquid transportation of microfluidic sweat sensors. Macromol. Rapid Commun..

[30] Huang H.Z., Yuan Q., Yang X.R. (2004). Preparation and characterization of metal-chitosan nanocomposites. Colloid Surf. B Biointerfaces.

[31] Liu P.S., Chen Q., Liu X., Yuan B., Wu S.S., Shen J., Lin S.C. (2009). Grafting of zwitterion from cellulose membranes via ATRP for improving blood compatibility. Biomacromolecules.

[32] Xu M., Ji F., Qin Z.H., Dong D.Y., Tian X.L., Niu R., Sun D., Yao F.L., Li J.J. (2018). Biomimetic mineralization of a hydroxyapatite crystal in the presence of a zwitterionic polymer. Crystengcomm.

[33] Wu R.J., Li L., Dong G.L., Qin Y.Q., Li M.W., Hao H. (2022). Fabrication and characterization of zwitterionic coatings with anti-oil and anti-biofouling activities. J. Macromol. Sci. Part B Phys..

[34] Shen X., Liu T., Xia S.B., Liu J.J., Liu P., Cheng F.X., He C.X. (2020). Polyzwitterions grafted onto polyacrylonitrile membranes by Thiol- Ene click chemistry for oil/water separation. Ind. Eng. Chem. Res..

[35] Elgamouz A., Kawde A., Alharthi S.S., Laghoub M., Miqlid D., Nassab C., Bajou K., Patole S.P. (2022). Cinnamon extract’s phytochemicals stabilized Ag nanoclusters as nanozymes “peroxidase and xanthine oxidase mimetic” for simultaneous colorimetric sensing of H_2_O_2_ and xanthine. Colloid Surf. A Physicochem. Eng. Asp..

[36] Wang Y., Cheng C., Ma R.F., Xu Z.R., Ozaki Y. (2022). In situ SERS monitoring of intracellular H_2_O_2_ in single living cells based on label-free bifunctional Fe_3_O_4_@Ag nanoparticles. Analyst.

[37] Laudenslager M.J., Schiffman J.D., Schauer C.L. (2008). Carboxymethyl chitosan as a matrix material for platinum, gold, and silver nanoparticles. Biomacromolecules.

[38] Shen X.L., Wu J.M., Chen Y.H., Zhao G.H. (2010). Antimicrobial and physical properties of sweet potato starch films incorporated with potassium sorbate or chitosan. Food Hydrocoll..

[39] Pilicer S.L., Wolf C. (2020). Ninhydrin revisited: Quantitative chirality recognition of amines and amino alcohols based on nondestructive dynamic covalent chemistry. J. Org. Chem..

[40] Rukmini N., Kavitha V.S., Devendra Vijaya K. (1981). Determination of ascorbic acid with ferricyanide. Talanta.

[41] Filiz B.C., Elalmis Y.B., Bektas I.S., Figen A.K. (2021). Fabrication of stable electrospun blended chitosan-poly(vinyl alcohol) nanofibers for designing naked-eye colorimetric glucose biosensor based on GOx/HRP. Int. J. Biol. Macromol..

[42] Georgelis N., Fencil K., Richael C.M. (2018). Validation of a rapid and sensitive HPLC/MS method for measuring sucrose, fructose and glucose in plant tissues. Food Chem..

[43] Sun S.H., Wang H., Xie J.P., Su Y. (2016). Simultaneous determination of rhamnose, xylitol, arabitol, fructose, glucose, inositol, sucrose, maltose in jujube (*Zizyphus jujube* Mill.) extract: Comparison of HPLC-ELSD, LC-ESI-MS/MS and GC-MS. Chem. Cent. J..

[44] Shrivas K., Naik W., Kumar D., Singh D., Dewangan K., Kant T., Yadav S., Tikeshwari, Jaiswal N. (2021). Experimental and theoretical investigations for selective colorimetric recognition and determination of arginine and histidine in vegetable and fruit samples using bare-AgNPs. Microchem. J..

[45] Yang Q.M., Xie C., Luo K., Tan L.B., Peng L.P., Zhou L.Y. (2022). Rational construction of a new water soluble turn-on colorimetric and NIR fluorescent sensor for high selective Sec detection in Se-enriched foods and biosystems. Food Chem..

[46] Zhou W., Wang Y.W., Yang F., Dong Q., Wang H.L., Hu N. (2019). Rapid determination of amino acids of nitraria tangutorum Bobr. from the Qinghai-tibet Plateau using HPLC-FLD-MS/MS and a highly selective and sensitive pre-column derivatization method. Molecules.

[47] Patle T.K., Shrivas K., Patle A., Patel S., Harmukh N., Kumar A. (2022). Simultaneous determination of B_1_, B_3_, B_6_ and C vitamins in green leafy vegetables using reverse phase-high performance liquid chromatography. Microchem. J..

[48] Yu M.Q., Wen R.Z., Jiang L., Huang S., Fang Z.F., Chen B., Wang L.P. (2018). Rapid analysis of benzoic acid and vitamin C in beverages by paper spray mass spectrometry. Food Chem..

[49] Bakhsh H., Palabiyik I.M., Oad R.K., Qambrani N., Buledi J.A., Solangi A.R., Sherazi S.T.H. (2022). SnO_2_ nanostructure based electroanalytical approach for simultaneous monitoring of vitamin C and vitamin B_6_ in pharmaceuticals. J. Electroanal. Chem..

[50] Rodriguez-Emmenegger C., Houska M., Alles A.B., Brynda E. (2012). Surfaces resistant to fouling from biological fluids: Towards bioactive surfaces for real applications. Macromol. Biosci..

[51] Rodriguez-Emmenegger C., Schmidt B., Sedlakova Z., Subr V., Alles A.B., Brynda E., Barner-Kowollik C. (2011). Low temperature aqueous living/controlled (RAFT) polymerization of carboxybetaine methacrylamide up to high molecular weights. Macromol. Rapid Commun..

