# Supplementary material for: Parallel Monitoring of Glucose, Free Amino Acids, and Vitamin C in Fruits Using a High-Throughput Paper-Based Sensor Modified with Poly(carboxybetaine acrylamide)
Source: Biosensors (Basel). 2023 Nov 28;13(12):1001. doi: 10.3390/bios13121001 (PMC10741689; doi:10.3390/bios13121001)
Supplement: Supplementary file 1 [file biosensors-13-01001-s001.zip › biosensors-2706512-supplementary.pdf]

biosensors

Supplementary Materials

Parallel Monitoring of Glucose, Free Amino Acids, and Vitamin C in Fruits Using a High-Throughput Paper-Based Sensor Modified with Poly(carboxybetaine acrylamide)

Xinru Yin [1,†], Cheng Zhao [1,2,†], Yong Zhao [1,*] and Yongheng Zhu [1,*]

[1] College of Food Science and Technology, Shanghai Ocean University, Shanghai 201306, China; m210311019@st.shou.edu.cn (X.Y.); allenz_1222@foxmail.com (C.Z.)
[2] Henan Railway Food Safety Management Engineering Technology Research Center, Zhengzhou Railway Vocational & Technical College, Zhengzhou 451460, China
* Correspondence: yzhao@shou.edu.cn (Y.Z.); yh-zhu@shou.edu.cn (Y.Z.); Tel.: +86-15692165928 (Y.Z.); Tel.: +86-15000137862 (Y.Z.)
† These authors contributed equally to this work.

Citation: Yin, X.; Zhao, C.; Zhao, Y.; Zhu, Y. Parallel Monitoring of Glucose, Free Amino Acids, and Vitamin C in Fruits Using a High-Throughput Paper-Based Sensor Modified with Poly(carboxybetaine acrylamide). *Biosensors* **2023**, *13*, 1001. https://doi.org/10.3390/bios13121001

Received: 24 October 2023
Revised: 18 November 2023
Accepted: 20 November 2023
Published: 28 November 2023

Copyright: © 2023 by the authors. Submitted for possible open access publication under the terms and conditions of the Creative Commons Attribution (CC BY) license (https://creativecommons.org/licenses/by/4.0/).

Materials and Methods

Grafting pCBAA onto cellulose paper surface

According to the previous experience [1,2] , the grafting method was divided into four steps (Figure S1). (I) Synthesis of carboxybetaine acrylamide (CBAA) monomer: Under nitrogen protection conditions, 0.99 g of β-propiolactone was dissolved in anhydrous acetone with vigorous stirring. The solution appeared pale yellow. The reaction system was maintained at 0 °C. Then, 1.5 g of DMAPA was added dropwise during the reaction, resulting in the formation of a white precipitate. The reaction was carried out under nitrogen protection conditions for 5 hours. After the reaction, the mixture was filtered at 0 °C. The crude product was washed with anhydrous acetone and anhydrous ether. Then, it was vacuum-dried and stored in a refrigerator at 4 °C. The reaction yield was 81%. (II) Synthesis of ATRP initiator: Bromoisobutyryl bromide (0.41 mL, 3.34 mmol) was slowly added to a solution of mercaptoundecanol (0.75 g, 3.67 mmol) and pyridine (0.27 mL, 3.34 mmol) in anhydrous dichloromethane with stirring at 0 °C. The reaction mixture was stirred for 1 hour at 0 °C, followed by continuous stirring at room temperature for 16 hours. After the reaction, water was added to the reaction mixture, and the product was extracted with toluene. The organic extract was then washed with ether and saturated ammonium chloride solution, followed by drying with sodium sulfate. (III) Immobilization of initiator on CF surface: Firstly, the CF substrate was immersed in a solution of TEA (148 mg, 1.46 mmol) and catalyst DMAP in THF (20 mL). Then, BIBB (305 mg, 1.33 mmol) was added for the reaction. The reaction was carried out overnight at room temperature on a shaker. After the completion of the reaction, the filter paper substrate was rinsed with EtOH and THF to remove unbound polymers and by-products. Finally, the filter paper was dried overnight in a vacuum oven at 45 ℃. (IV) Grafting of CBAA onto cellulose surface using SI-ATRP: 28.533 mg of CuBr and 61.707 mg of BPY, along with the CF chip coated with the initiator, were sealed together in a reaction tube under nitrogen protection conditions. Then, 1 mL of deoxygenated methanol was added to this reaction tube. In another reaction tube, 600 mg of CBAA monomer was dissolved in deoxygenated methanol (2.2 mL) and water (0.8 mL). The mixture was transferred to the previous reaction tube under nitrogen protection conditions using a deoxygenated syringe. The mixture was reacted under nitrogen protection conditions. After the completion of the reaction, pCBAA was taken out from the reaction tube and washed with ethanol, PBS, and distilled water. Then, it was vacuum-dried and stored for later use.

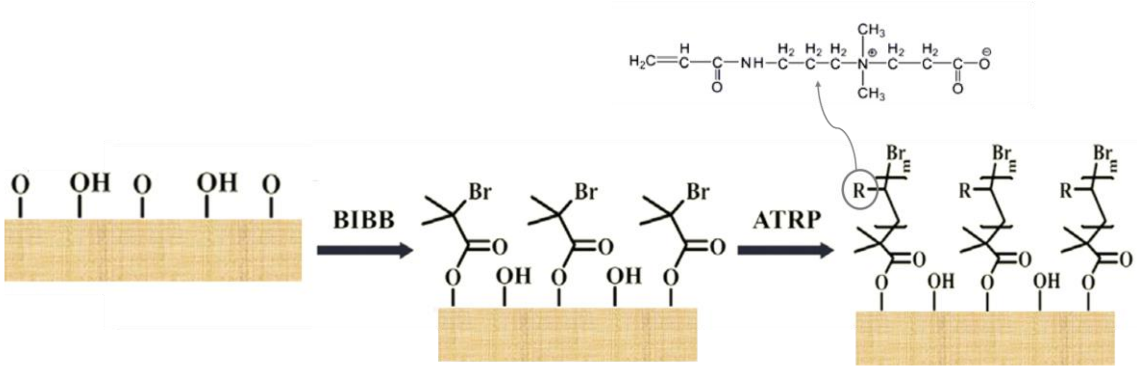

Figure S1. Reaction scheme for grafting pCBAA onto CF.

Detection methods of glucose, free amino acids, and vitamin C with pCBAA-μPAD

Glucose detection

Firstly, in the detection zone of the pCBAA-μPAD, 1 μL of glucose standard solution or an actual fruit sample was added, followed by 1 μL of 1.5 mg/mL glucose oxidase. Next, 1.5 μL the prepared colorimetric reagent mixture (1 mL of acetate-sodium acetate buffer at pH=3, 0.2 mol/L, 125 μL of 100 mmol/L TMB solution, 125 μL of 40 mg Ch-Ag NPs/L Ch-Ag NPs) was added, where the solvent for the TMB solution was acetone. The sensor surface was covered with sealing film and placed in a 37 °C oven for 30 minutes for color development. After color development, the color change was captured using a camera, and the grayscale values were analyzed using Image J software.

Free amino acid detection

In the detection area of the pCBAA-μPAD, 1 μL of amino acid standard solution or actual fruit sample, 1 μL of acetate buffer solution at pH=5, and 1 μL of 1.5% indigo trione were sequentially added. The solvent for indigo trione was ethanol. The sample was placed in a 60°C oven for 5 minutes for color development. After color development, the color change was captured using a camera, and the grayscale values were analyzed using Image J software.

Vitamin C detection

In the detection zone of the pCBAA-μPAD, 1.5 μL of vitamin C standard solution (1-10 mmol/L) or actual fruit sample, 1.5 μL of color reagent (100 mmol/L ferric chloride solution, 0.5% $K_3[Fe(CN)_6]$ solution, and

12 mol/L HCl in a ratio of 3:2:1) were sequentially added. After color development, the color change was captured using a camera, and the grayscale values were analyzed using Image J software.

Image processing and data acquisition

The photos were captured by a digital camera fixed on a simple tripod. The camera was operated in automatic mode at a distance of 20 cm from the paper device. Images were taken both before and after exposure to the nutrients. Color intensity changes were calculated using Image J software, which converted the captured images into numerical mean values for red, green, and blue color elements. The difference between the color values of the before and after photos was calculated using the following equation:

$$\Delta R = \bar{R}_{after} - \bar{R}_{before} \quad (S1)$$

$$\Delta G = \bar{G}_{after} - \bar{G}_{before} \quad (S2)$$

$$\Delta B = \bar{B}_{after} - \bar{B}_{before} \quad (S3)$$

In this equation, the difference values of red, green, and blue color elements are denoted by ΔR, ΔG, and ΔB, respectively. Next, the difference values between the reaction point and the blank point were re-converted to gray intensity by the following equation:

$$\Delta Gray = 0.30\Delta R + 0.59\Delta G + 0.11\Delta B \quad (S4)$$

Based on the above formula, the mean relative intensity was obtained from the RGB readings measured in the reference area and sensing area after each experiment was repeated three times.

Statistical data analyses were performed in the Microsoft Excel software package (Microsoft, Redmond, WA, USA) and Origin 2019b (OriginLab, Northampton, MA, USA).

XPS Analysis

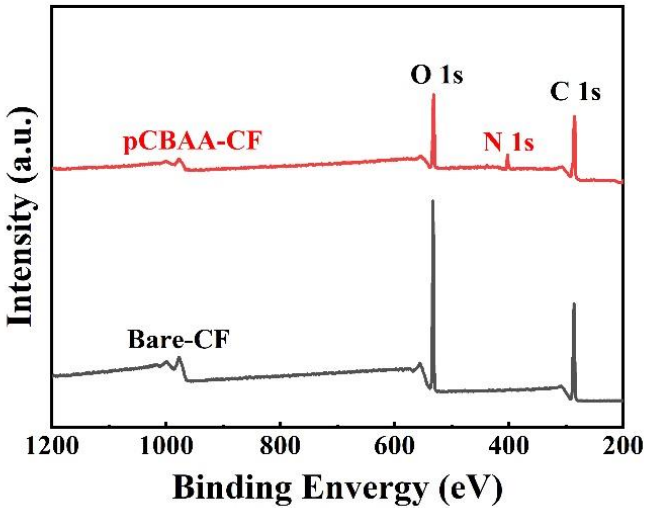

Figure S2. The XPS spectra of bare-CF and pCBAA-CF.

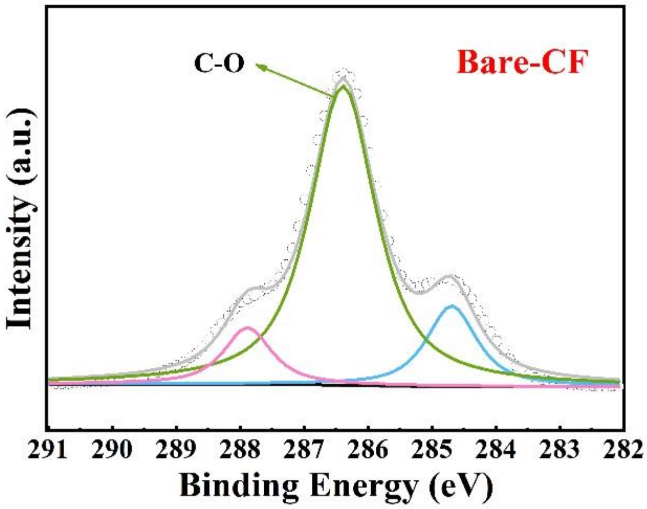

Figure S3. Representative XPS high-resolution C1s spectra of bare-CF.

Synthetic Ch-Ag NPs

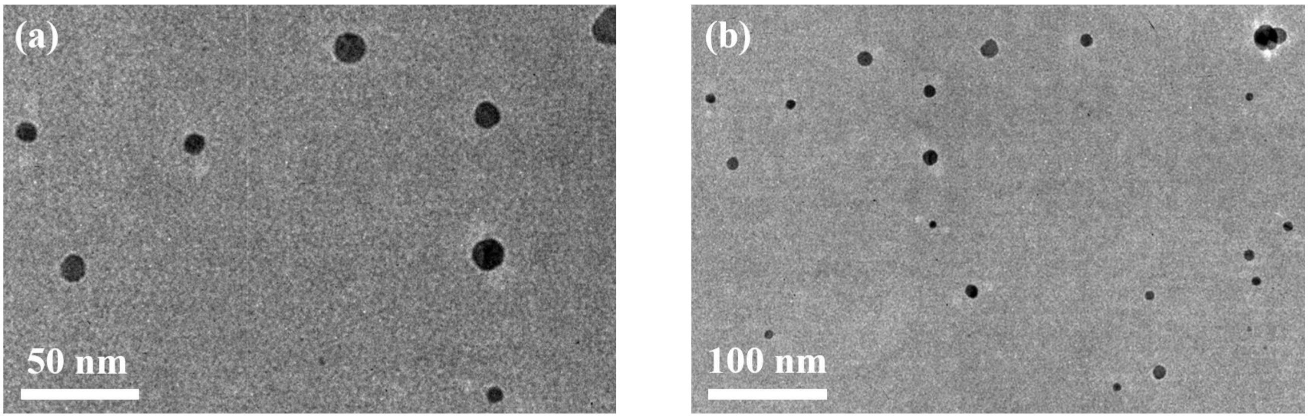

Figure S4. TEM image of Ch-Ag NPs.

References

1. Rodriguez-Emmenegger, C.; Houska, M.; Alles, A.B.; Brynda, E. Surfaces resistant to fouling from biological fluids: Towards bioactive surfaces for real applications. *Macromol. Biosci.* **2012**, *12*, 1413-1422.
2. Rodriguez-Emmenegger, C.; Schmidt, B.; Sedlakova, Z.; Subr, V.; Alles, A.B.; Brynda, E.; Barner-Kowollik, C. Low temperature aqueous living/controlled (RAFT) polymerization of carboxybetaine methacrylamide up to high molecular weights. *Macromol. Rapid Commun.* **2011**, *32*, 958-965.
